# An Exploratory Survey of Mental Health Social Work in Europe

**DOI:** 10.3390/ijerph181910462

**Published:** 2021-10-05

**Authors:** Kevin Stone, Pearse McCusker, Gavin Davidson, Sarah Vicary

**Affiliations:** 1 School of Health Professions, Faculty of Health, Drake Circus Campus, Plymouth University, Plymouth PL4 8AA, UK; 2 School of Social and Political Science, University of Edinburgh, Edinburgh EH1 2QL, UK; pearse.mccusker@ed.ac.uk; 3 School of Social Sciences Education and Social Work, Faculty of Arts Humanities and Social Sciences, Queen’s University Belfast, Belfast BT7 1NN, UK; g.davidson@qub.ac.uk; 4 School of Health Wellbeing and Social Care, Faculty of Wellbeing Education and Languages, The Open University, Walton Hall, Milton Keynes MK7 6AA, UK; sarah.vicary@open.ac.uk

**Keywords:** social work, mental health, Europe, involuntary admission

## Abstract

This article reports on an exploratory study comparing mental health social work (MHSW) in Europe. There has been very limited previous research comparing approaches to MHSW in Europe and so the aim of the study was to develop a better understanding of the similarities and differences between and, where relevant, within countries (referred to as jurisdictions). An online survey was distributed mainly through existing European networks and social media to seek information on the role, nature, extent and context of MHSW in a range of European jurisdictions. Conducted during the COVID-19 pandemic, there were 158 responses from 10 jurisdictions. Data were analyzed using thematic analysis. From this analysis, four main themes were identified, relating to: role; law, policy and education; the distinctive contribution made by MHSW; and the key challenges for MHSW. The study demonstrates that MHSW, although it is described and provided in different ways and is confined by a range of factors, plays an important role in mental health services across jurisdictions. There are also interesting differences between contexts, especially in the balance of therapeutic, legal and specialist/generic approaches, some of which reflect the World Health Organisation’s vision and objectives for mental health. Lastly, the study illustrates a need and provides a valuable basis for further comparative and collaborative work to define MHSW and enhance the contributions it makes.

## 1. Introduction

Social work is an international profession with an agreed global definition, which specifies that it is:

‘A practice-based profession and an academic discipline that promotes social change and development, social cohesion, and the empowerment and liberation of people’ [1].

(p. 1)

Despite this shared definition, the International Federation of Social Work (IFSW) goes on to acknowledge that there may be variations in emphasis in each country, or even among regions within countries (hereafter referred to in this article as jurisdictions). The variations in emphasis may be historical, cultural and social, and therefore these contexts are necessary to understand in order to consider social work in that setting. It follows, then, that care should be taken in directly comparing social work within and between jurisdictions, and this article discusses an exploratory study which sets out to do so. The aim is to stimulate and inform the consideration of potential developments within social work practice and to share emerging ideas and concepts intra- and internationally.

As a profession, social work’s engagement with mental health is poorly understood and under-researched and has gained little attention in European policy. Where it does, social work is recognised as intervening within mental health services to engage with ‘social’ aspects in the form of perspectives, theory, values and practices to complement those of allied health professionals. The World Health Organisation’s Mental Health Action Plan (MHAP) 2013–2020 (extended to 2030) identifies social, economic, cultural and political factors as social determinants of poor mental health, indicating that it is caused and maintained by factors including poverty, isolation, exclusion, discrimination and stigmatisation which the MHAP identifies as key objectives for change [2]. With its emphasis on promoting ‘…social change and development and social cohesion…’ social work arguably has a key role as a discipline in contributing to MHAP’s objectives [1] (p. 1). Moreover, the profession’s values and aims in respect to social justice are encapsulated in the WHO’s broader vision for change:

‘A world in which mental health is valued, promoted, and protected, mental disorders are prevented and persons affected by these disorders are able to exercise the full range of human rights and to access high-quality, culturally appropriate health and social care in a timely way to promote recovery… participate fully in society and at work free from stigmatization and discrimination’ [2].

(p. 34)

A critical factor for the success of any health and social care infrastructure is the distribution of competent skilled health and social care workers who have a clear understanding of their professional roles and the resources to effect meaningful change [2] (p. 15) [3]. The study upon which this article is based seeks to provide initial and emerging insights into ways in which the role of mental health social work (MHSW) is understood, enacted and supported in a range of jurisdictions across Europe. It will explore the degree to which MHSW can be seen to contribute to the WHO’s vision and the objectives which underpin this.

## 2. Literature Review

A number of frameworks have been proposed to manage the complexity of the comparison of social work between jurisdictions. An early and influential one by Lorenz, with a focus on developments in Europe, highlighted the importance of considering social work in the context of the relevant welfare model or regime, ideological and academic discourses and wider social movements [4]. Later, Lorenz highlighted the historical and ongoing processes of both the diversification and convergence of approaches to social work in Europe and acknowledged both the challenges for comparative analysis presented by the wide range of terms, roles and structures that have developed, and the potential benefits of international connection and cooperation across social work education, research, policy and practice [5]. Meanwhile, Houston and Campbell suggested a further framework which considered three main social domains: macro (large-scale international social processes that have an indirect impact on social work, such as changes to the European Union or international conflict); mezzo (which includes the relationships between the state, the wider welfare context and social work); and micro (where approaches to everyday social work practice are the focus) [6].

Continuing Lorenz’s earlier discussion of whether it is reasonable to identify a distinctive model of European social work or if the diversity of contexts and approaches are more appropriately discussed as social work in Europe, Spratt et al., in their comparison of child protection in five European countries, reported that although it was important to consider context and culture, beyond surface variations, there was a remarkable consistency in the issues and ideals involved, which further reinforced the potential benefits of international comparisons [7]. More recently, Kessl et al. have presented an overview of social work in Europe, which explores the macro social narratives, including neo-liberalism and risk; some of the main areas of social work practice although not mental health; some developments in theoretical and methodological discourses such as evidence-based practice and transnational social work; and some speculation about possible future developments such as professionalisation processes and the potential for a re-emergence of community work [8].

As indicated above, a key focus of social work in Europe concerns people with mental health problems, although there has been relatively little research on social work’s role or on the comparison of approaches. Mental distress is relevant to all aspects of social work, which should be reflected in pre- and post-qualifying training [9] but the focus here is on social workers whose role is specifically to work with people who are experiencing mental health problems. Morrison and Davidson have suggested that social work provides a distinctive contribution to mental health services, which includes relationship based approaches; systemic practice; a focus on social determinants and social wellbeing; additional and alternative perspectives to more individualised and clinical approaches; an emphasis on social justice; the promotion of human rights; anti-oppressive practice which addresses the wider issues of stigma, discrimination and power; community development; legal expertise; and a key role in safeguarding [10].

Based on interviews with mental health social workers in England. Tucker and Webber reported a number of key themes in the role, including statutory duties; the recovery approach; working with complexity; working with communities; leading work under mental health and mental capacity law; working across boundaries; providing a holistic perspective; and prioritising client needs [11]. Since the introduction of specific social work roles under mental health law, there has been a debate about the impact of these statutory duties on the mental health social work role [12,13]. Abendstern et al. explored multi-disciplinary perceptions of the role of social work in mental health services in England and highlighted social work’s role in promoting the service users’ voice and in implementing the social model [14]. In exploring service users’ views of social work in mental health services, Boland et al. suggested that there may be less of a clear understanding of social work’s distinctive role [15].

The rationale for the study which this article discusses was therefore based on a number of drivers:

- the potential general benefits of international comparisons for understanding and development; the previous frameworks and comparisons in Europe reinforced this potential and a key issue identified was the relative lack of previous work focused on MHSW.
- the context of the COVID-19 pandemic appears to have raised awareness of the central importance of mental health and so there is an opportunity to influence how the increased prioritisation of mental health support may be implemented.
- to help support and inform the newly established MHSW Special Interest Group as part of the European Social Work Research Association This includes exploring if social workers do indeed bring a ‘social perspective’ and what this might contribute to mental health—evidence that would be of interest to a wider, multidisciplinary audience.

Finally, in relatively uncertain times, especially in the context of Brexit, there was perhaps also an underlying desire to connect and work with colleagues in other European countries to try to inform and influence positive change in this specialism.

## 3. Materials and Methods

The study used a qualitative online survey to explore how MHSW is conceptualised, practised and understood across European jurisdictions. Designed specifically for this study, the survey included 11 open and 4 closed questions, starting by asking where social workers were geographically located, followed by a series of questions about role and context regarding mental health social work practice, including statutory registration arrangements, legal frameworks and theoretical and practical models. The survey was distributed on one occasion across continental Europe and remained open for an eight-month period. Distribution methods included established European communication channels, social media and direct contact with higher education institutions in Europe.

The survey achieved 191 responses of which 33 were non-completions (in which the participants did not move beyond the consent page). Of the remaining 158, 76 completed the survey, with the rest providing responses to one or more of the questions. Responses came from ten different jurisdictions; for six of these there were two or fewer respondents. UK jurisdictions included Scotland, Northern Ireland and England, but not Wales (Scotland *n* = 39; England *n* = 3; Northern Ireland *n* = 2). Five respondents identified themselves as being located in the UK with no specific jurisdiction/nation identified. Croatia and the Republic of Ireland numbered 23 each, whereas a single response was received from Albania, Austria, Sweden, Switzerland and Turkey. The response rate was high from 3 jurisdictions, namely Scotland (25%), the Republic of Ireland (15%) and Croatia (15%). Reasons for the low response rate from others are not clear but may reflect particular factors such as survey fatigue (for example, mental health social workers in England had only recently been asked to complete a separate survey) and challenges in disseminating the survey. The level of detail given in answers was variable.

All subjects gave their informed consent for inclusion before they participated in the study. The study was conducted in accordance with the Declaration of Helsinki, and the protocol was approved by the School of Social and Political Science Research Ethics Committee, University of Edinburgh (266802 24-1-20). At the beginning of the survey, participants were asked to read an information sheet, data privacy notice and consent form. Without consent being given, participants could not proceed with answering the survey questions. Participants remained anonymous unless they opted to provide contact details to join a mental health special interest group. Data were analysed and coded separately by two authors (two and four) utilising an inductive thematic analysis [16], who then came together to agree on the broad themes and sub-themes. This was subsequently shared and agreed upon by all authors.

## 4. Results

Analysis of the data revealed emerging yet distinct similarities and differences in MHSW across jurisdictions. These have been collated into four themes: role; law, policy and education; distinctive contributions made by MHSW; and key challenges facing MHSW. Each of these are discussed in turn; respondents’ quotes are identified by jurisdiction and respondent number.

### 4.1. Role

In the countries represented in the survey, the data suggest that MHSW exists on a continuum, linked to factors including law, policy and education (discussed below) and the degree to which mental health services are established and resourced. Within this, respondents indicated a wide variety of roles undertaken by social workers in relation to mental health, but broadly speaking these sat within two main categories. The first relates to legally mandated roles, exclusively or predominantly for social workers, around the use of the compulsory care and treatment of people with mental health issues, as is the case in England, Northern Ireland and Scotland. Here, answers were characterised by the details of prescribed roles and duties under the relevant legislative frameworks, such as:

“…role under the Mental Health act to detain service users who need professional support.”

(England, 182)

“Application of MH legislation.”

(Northern Ireland, 8)

“…coordination [of] involuntary admission, overseeing guardianship orders.”

(Scotland, 70)

It also included working closely with medical/health colleagues and a key responsibility for upholding human rights in relation to involuntary admission to hospital:

“…specially qualified to undertake joint assessments with Psychiatrists re: involuntary admissions and ensuring patient rights under legislation.”

(Scotland, 84)

In countries where there is a known role for social workers identified in legislation, the use of language tended to echo this formal or procedural role. Examples included:

“…criteria met for detentions, case management, attendance at tribunal.”

(Scotland, 74)

“Assessment of social care needs, role under the Mental Health act to detain service users who need professional support.”

(England, 182)

Respondents from the other jurisdictions indicated either that social workers were less involved or not involved in such processes, that such laws did not exist or did not refer to them at all.

The second main category was social workers who worked directly with people with mental health issues but with a less defined mental health role. This work took place in a range of settings, including community mental health teams, social welfare offices, general hospitals and mental health hospitals, retirement homes and forensic settings, including prisons. There was considerable overlap in how respondents described their functions. Consistent themes were assessment and care management, developing care packages for hospital discharge planning, and supporting people to access housing and other community and social welfare resources, as seen here respectively for the Republic of Ireland, Croatia and Turkey:

“…working as MHSW [mental health social workers] we do the following—psychosocial assessments, discharge planning, housing, welfare, support re abuse, safeguarding, family issues, legal rights, psychoeducation.”

(Republic of Ireland, 14)

“Determining patient’s social status, collecting data, determining their social networks and family support, conducting counselling and psychotherapy, providing information about their rights within the social welfare system, coordinating treatment plans and providing housing.”

(Croatia, 128)

“When social workers work in the field of mental health, they usually work as a member of the mental health team and contribute by conducting assessment (via home and school visits), work on housing issues, coordinate social services, bridging between the clinic and family and community.”

(Turkey, 180)

Sweden, by contrast, was something of an outlier, with MHSW being split into two contrasting roles: one located in psychiatric hospital settings, where the main role was discharge planning, and the other in municipal settings in a specialised branch referred to as “social psychiatry”, typically working as managers of agencies addressing people’s needs rather than working directly with them.

A number of notable sub-themes regarding roles emerged. The first conveyed differences in how respondents characterised the role of MHSW, as either procedural (predominantly care management) or therapeutic in nature. In two locations where the role under legislation is not yet formalised, MHSW is described in terms of psychotherapeutic interventions, with social work itself seen as an intervention:

“housing, welfare, rehabilitation, counselling, working on family relationships”

(Republic of Ireland, 10)

“Advocacy, care management, case management, housing, access to service, therapeutic interventions e.g., counselling, family work, bereavement work”

(Republic of Ireland, 15)

“Determining patient’s social status, collecting data, determining their social networks and family support, conducting counselling and psychotherapy, providing information about their rights within the social welfare system, coordinating treatment plans and providing housing”

(Croatia, 128)

The second sub-theme centred on concerns about a lack of attention being paid to social work in the context of the mental health field. It included the perceived shortcomings of not having a more defined mental health social worker role. This was reflected in some comments from respondents in counties with established mental health systems, such as England:

“In England, MHSW is not well defined—there is an obligation on local authorities to provide a MHSW workforce, and an expectation that MHSW will form part of the multidisciplinary environment in mental health, but the scope and intention of that role is not defined, and definitions have therefore emerged which are very localised and contextual, in response to local working arrangements. Recent attempts to change this in policy have had minimal impact on the frontline of practice, where the corresponding health roles are much more clearly delineated. The increasing withdrawal of social workers from the multidisciplinary environment has seen a shift toward social work in mental health becoming a statutory role linked to the local authorities’ legal obligations, while those social workers who remain within health settings tend to fall into distinctly more generic practitioner roles.”

(England, 181)

It was also evident in others, where mental health services were described as limited or developing, such as Albania:

“Although social workers are listed as part of the professionals engaged with psychosocial services, no specific actions target social work/ers per se.”

(Albania, 65)

### 4.2. Law, Policy and Education

In line with the above findings, the analysis revealed distinct differences across countries in terms of the legal and policy frameworks for mental health as a whole and the provision of education and opportunities for social workers wishing to work in this area. Again, a key dividing line was if countries had roles for mental health social workers that were formalised by legislation. In these jurisdictions (England, Northern Ireland and Scotland) respondents’ answers showed consistency in the levels of knowledge about mental health and capacity legislation; for example, respondents from Northern Ireland noted the key legislation—the Mental Health (NI) Order, Mental Capacity Act (Northern Ireland) 2016—that defines the mandated mental health social worker role (Approved Social Worker). Many also named generic social work and other legislation that they felt had an influence on MHSW. This included safeguarding legislation, such as the Care Act 2014 in England, the Adult Support and Protection (Scotland) Act 2007, the Human Rights Act 1998 and laws designed to enhance support services for people with mental health issues and their carers, for example, the Social Care (Self-Directed Support) (Scotland) Act, 2014 and the Carers (Scotland) Act, 2016.

Respondents from these countries were also cognisant of well-established policy frameworks for mental health, which social workers were obliged or encouraged to follow. These policies ranged from formal guidance relating to statutes in the form of Codes of Practice, to good-practice guides issued by national bodies, including the Department of Health and Social Care in England and the Mental Welfare Commission, Scotland. Some also referred to national mental health strategies and to broader policy that informed mental health social work and particular service user groups:

“Dementia strategy, Mental Health Strategy 2017–2027, suicide reduction and prevention, improving lives of those living with Autism and/or learning disability.”

(Scotland, 93)

Although the Republic of Ireland’s mental health law does not set aside a specific role for social work, the responses pointed to a range of related law and policy that social workers followed, including the Assisted Decision Making (Capacity) Act 2015, the Children First Act 2015 and Sharing the Vision: A Mental Health Policy for Everyone, 2020 [17]. In addition, one person advised that a “Specialist Interest Group for Social Workers in Adult Mental Health” had been established and had developed a guidance document. Although social workers are not required to follow this guidance, its emergence signals a perceived need for more discussion around and support and direction for the MHSW role in this country.

In contrast, respondents from other countries either noted emerging or limited mental health legal and policy frameworks or did not provide answers. In Croatia, in addition to generic social welfare law, most respondents referred to the Law on the Protection of Persons with Mental Disorders, introduced in 2015, a significant aim of which was to improve protections surrounding involuntary admissions to hospital [18]. In Sweden, the respondent referred to generic acts “The Health Services Act” and “the Social Service Act”. In Austria, the answer suggested there were “no special [mental health] laws”, whereas for Turkey, the comment pointed to an ongoing and lengthy delay in the development of mental health legislation following the agreement of a mental health policy in 2006.

A similar pattern in respect to the education of the workforce emerged, in that where jurisdictions do have a recognised statutory role there appears to be a robust training and education process in place for this, as illustrated by England, Scotland and Northern Ireland. In jurisdictions without this, the provision of education and training appeared more varied, ad hoc or limited. In the Republic of Ireland, respondents advised that social workers could undertake training required to perform an equivalent statutory ‘Authorised Officer’ role, but another reported that mental health as a subject was poorly taught, indicating a need for a state-regulated system of accredited social work post-qualifying programmes; the latter was mirrored by comments from Croatian respondents. In Sweden, the answer indicated that no specific mental health qualification was required for MHSW roles in municipal services, but there was a “recently made specialization university-level curriculum that is mandatory for social workers in medical/psychiatric organizations”.

Some respondents also commented on the level of foundational mental health knowledge required for the generic social work degree or equivalent qualification. In the Republic of Ireland, this included:

“…mandatory training requirements set out by the Mental Health Commission for all staff … Mental Health Act, Basic Life Support, Manual Handling and Therapeutic Management of Violence and aggression.”

(Republic of Ireland, 19)

In addition, a number of responses referred to dual psychological or psychotherapeutic qualifications held by social workers—including dialectical behavioural therapy and behavioural family therapy—which supported their roles as MHSW practitioners (Croatia, 128; Republic of Ireland, 2, 19, 20, 21).

### 4.3. Distinctive Contributions Made by MHSW

It was notable that many of the fullest answers provided in the survey focused on the distinctive contributions made by MHSWs in comparison to medical, allied health or other professionals. As such, this suggests that MHSWs see the role as unique and important despite the challenges they face (discussed below). There was a high degree of consensus among respondents about key defining features of their work. These centred on operating from social and/or psychosocial models of mental health, defined as holistic, social and rights-based in nature, and in contrast to medicalised approaches:

“Social workers take a different approach to mental health than that of our health colleagues. We are able to take a holistic approach rather than basing intervention solely on the medical model.”

(Scotland, 85)

“Social workers do not view the patient in a vacuum, they work systemically and highlight the many social issues that impact on the patient’s mental health.”

(Republic of Ireland, 16)

Frequent reference was made to empowerment and using strengths-based and recovery approaches:

“We consider all elements of a person’s life/experience and focus on the premise of empowering our clients. We also focus on their strengths and encourage self-determination and independence.”

(Republic of Ireland, 17)

“Their [MHSWs’] holistic view on recovery and focus on non-medical interventions to support recovery.”

(Scotland, 76)

Understanding the social impact of mental illness on people and a concomitant focus on addressing stigma and discrimination and providing information about rights also featured:

“Mental health social workers have to hold an understanding of how an individual’s poor mental health can have a direct impact on their daily living. Social workers tackle stigma and discrimination for clients within the community and within services.”

(Scotland, 83)

“Providing information about their rights within the social welfare”

(Croatia, 128)

“There is often a stigma in society about mental health and having dedicated social work practitioners helps to lessen this stigma and create community where it is needed most.”

(Scotland, 70)

The responses also indicated that without social workers key areas of need would not be addressed by other mental health professionals:

“No other disciplines are as involved in advocacy around Housing and Social Welfare issues as are Social Workers.”

(Republic of Ireland, 20)

“Social justice, nobody else does it.”

(Republic of Ireland, 15)

Some answers also pointed to a safety net provided by MHSW, which in part was related to having a more ‘catch-all’ and less narrowly defined role:

“Social workers are very much a jack of all trades and that is definitely seen within mental health social work. Anything that other agencies won’t do social workers will address that unmet need.”

(Scotland, 85)

“Social workers take a more holistic approach considering they have to contact the user, his family, various institutions, so they have more relevant information to work with, and are literally forced to fulfil many obligations in care for [the] user. So their approach is not [as] narrow as the one of other professionals.”

(Croatia, 126)

Lastly, one respondent provided a more nuanced reflection on the distinctive contribution made by MHSWs. This captures some of the values and aspirations expressed above but goes on to highlight barriers that can limit their expression in day-to-day practice. It also raises a question about how other professionals might view apparently ‘social-work-specific’ traits:

“Social workers in England work in a range of settings undertaking some distinctly different roles, which do not always overlap. I would suggest that what separates them from other mental health professionals is a more person-centred, holistic perspective, but I would also suggest that they are limited in their ability to work on these ways by the expectations of the organisations they work within, and the restrictions placed on the roles they can undertake (within a framework of statutory duties in local authorities, within a Care Programme Approach framework in health settings). I would also wonder whether other mental health professionals would take a different view of whether that holistic approach was a social work specific trait.”

(England, 181)

### 4.4. Key Challenges for Mental Health Social Work

The final theme elucidates and expands on the key challenges facing MHSW, some of which are intimated above. These fall into two main categories, albeit they are interrelated: the first describes the societal and resource problems encountered in working with people using mental health services; the second outlines professional/status barriers that respondents felt hindered their work.

There was a consensus about the range of societal factors that contribute to mental ill-health across jurisdictions, with some context-specific examples also provided. Perhaps unsurprisingly, poverty, unemployment, addiction, lack of access to housing and health resources and family breakdown were cited frequently:

“…poverty, unemployment, lack of service provision, poor rural transport links and a lack of housing.”

(Scotland, 81)

“Poverty, unemployment, health inequalities based socio-economic factors.”

(Republic of Ireland, 79)

The problems of stigma and discrimination were highlighted, as was specific reference to this in relation to work, education, immigration and asylum:

“Stigma, oppression and discrimination against people with mental health problems. A work market where people with a diagnosis of mental illness are all but excluded.”

(Scotland, 67)

“Racism, housing of refugees, discrimination against minority groups.”

(Ireland, 13)

“…being discriminated from the work and education system.”

(Turkey, 180)

Responses highlighted particular types of discrimination related to individual jurisdictions’ history and religious and ethnic mixes. For example, one response from Northern Ireland alluded to the lasting impact of the ‘Troubles’ on people’s mental health, in terms of:

“…sectarianism, racism, transgenerational trauma.”

(Northern Ireland, 6)

Similarly, respondents from Croatia reflected the legacy of the Balkan Wars and discrimination facing the Roma community:

“…post-war problems.”

(Croatia, 124)

“…issues concerning Roma population, … a large number of young retirees due to war.”

(Croatia, 128)

The issue of ‘Brexit’ also featured, but interestingly only in UK respondents’ answers. The following comments link it to xenophobia and fears about its long-term impact, including on human rights law:

“Discrimination linked to xenophobia in the context of Brexit is a potential issue in all areas, and Brexit remains a highly divisive social topic; the ramifications of Brexit, both psychological and practical, are also a concern.”

(England, 181)

“I fear that leaving the EU (and therefore the jurisdiction of the European Court of Human Rights), could have a seriously detrimental effect on liberty.”

(Scotland, 75)

Surprisingly few answers referred to the COVID-19 pandemic, but those that did highlighted a perceived impact on people’s mental health, as well as a fear about its longer-term consequences for resources:

“COVID-19 pandemic has, in my opinion, played a significant part in increasing fear, anxiety and paranoia.”

(Scotland, 75)

This mirrored a wider systemic problem of insufficient resources to support people experiencing mental ill-health, with retrenchments of service delivery and funding cuts evident in answers across jurisdictions:

“…cutbacks in welfare systems.”

(Sweden, 179)

“…services to support people to get back into work after an acute illness have been decimated, literally.”

(England, 67)

“There is a lack of a support network in the local environment after the completion of hospital treatment, therefore many patients are being re-hospitalized.”

(Croatia, 128)

“Demand is increasing for mental health social workers and worker’s caseload are high. Specialised community services for mental health are low in resources.”

(Scotland, 83)

As indicated, problems with the professional status of MHSW were also identified as a key challenge across jurisdictions. There was a sense among some respondents that MHSW was at the periphery of mental health services and that it was poorly understood:

“Social workers in mental health are not accepted and appreciated profession in mental health domain.”

(Croatia, 122)

“I don’t think people fully understand the role of mental health social work in this country.”

(Scotland, 85)

Furthermore, respondents commented on the significance of social workers having less power and their professional opinions counting for less than their medically trained colleagues. This reflected a view around the dominance of medical model approaches, although it was recognised by some that psychiatrists increasingly operate from more integrated perspectives:

“Social workers are currently struggling to stay as a mental health profession… The system turns to a more medically dominated field where psychiatrists and clinical psychologists become dominant and therefore social aspects of the recovery is neglected.”

(Turkey, 180)

“It [MHSW] has come a long way in recent years but social workers still must often concede to colleagues from the health professions—mostly psychiatrists. Despite generally good interdisciplinary working, psychiatrists and other doctors often have the final say in the way someone will be treated and are viewed by people using services and their carers as the ‘leader’ of any team.”

(Scotland, 76)

“…we still have to battle against the medical model, we have a strong network of SW managers in MH and we provide peer support to each other and try to develop the SW profession within MHS. it remains an under-resourced area, no access to training budgets, staffing shortages.”

(Republic of Ireland, 19)

Some respondents commented on attempts to improve the professional status of MHSW. The complexities of status were, however, reflected in a number of conflicting answers. For example, in the Republic of Ireland, one social work manager spoke of tangible progress having been made:

“In Child and Adolescent Psychiatry Social Work is very valued and it enjoys a high status. In Adult Mental Health Services, the Medical Model continues to be quite dominant and Social Workers have to fight hard to forge a role. Since 2010/2012 significantly more Social Workers have been employed in Adult Mental health Services as a result of ‘Vision for Change’. As Social Work Managers we are collaborating more with each other across the country to have greater uniformity in the development of the role.”

(Republic of Ireland, 20)

In contrast, two other respondents from the Republic of Ireland offered different views:

“[MH] Social work is underused, under respected and under resourced. Upper management always describe their admiration when they sit in on work, we are doing but refuse to support Social Work as a profession when it comes to upskilling for example.”

(Republic of Ireland, 15)

“I previously worked in California in the US and there was a specialist training and accreditation for social workers in mental health (LCSW) which I feel gave more status and therefore more influence in the mental health system. I think that social work is not viewed in the same way in Ireland and is often seen as more of a medical social work model and social workers are not viewed as skilled mental health professionals.”

(Republic of Ireland, 21)

Lastly, some respondents offered suggestions on how the professional role and status of MHSWs could be better recognised and further developed. In the main, this centred on improving educational and training opportunities, in addition to incentivising social workers to enter the field:

“There needs to be more of an incentive for workers to undertake the training, so as to increase the workforce, as it will be difficult to sustain this level of workload pressure.”

(Scotland, 110)

“…it could be improved by the introduction of formal training in mental health social work.”

(Republic of Ireland, 18)

## 5. Discussion

Our findings indicate that MHSW is practiced in a variety of ways across Europe, as per the IFSW definition above, and resonates with Lorenz’s view that social work needs to be observed in the context of the ‘relevant welfare model or regime, ideological and academic discourses, and wider social movements’ [4]. This variation of approach and discourse can be a strength for MHSW, but this depends firstly on MHSW being adequately resourced and recognised, and secondly on building an appropriate infrastructure for knowledge exchange and learning from good practice across jurisdictions. Overall, the data illustrate the important contribution made by MHSWs in their jurisdictions and specific contexts. This rests particularly in acknowledging and responding to the social determinants of mental health and the impact on the social spheres of people’s lives and efforts at micro-levels to achieve social justice. Respondents were clear that other professions do not address these aspects to the degree that MHSW does, or even at all. In this, MHSW appears to be reflecting social narratives that are critical of neoliberal economic policy, with its resultant impact on poor mental health and wellbeing [9].

The responses from participants in this study also echo Morrison and Davidson’s description of MHSW’s contribution to mental health services, combining relationship-based approaches; systemic practice; a focus on social determinants and social wellbeing; additional and alternative perspectives to more individualised and clinical approaches; an emphasis on social justice; the promotion of human rights; anti-oppressive practices which address the wider issues of stigma, discrimination and power; community development; legal expertise; and a key role in safeguarding [11]. The findings also highlight MHSW’s use of social or psychosocial theory and practice to complement, and at times balance, more individual and bio-medical, pharmacological approaches, suggesting that MHSW is well-positioned to highlight the social determinants of mental health problems, and on preventative approaches through its holistic perspective and emphasis on the role of the systemic context of individuals. On these issues, there was a striking similarity in the respondents’ views [7].

The survey data also suggest that MHSW can and does make a contribution to enable the World Health Organisation’s 2013–2020 vision for mental health to be realised. What follows is a discussion using the objectives of that vision, namely:

1. to strengthen effective leadership and governance for mental health;
2. to provide comprehensive, integrated and responsive mental health and social care services in community-based settings;
3. to implement strategies for promotion and prevention in mental health;
4. to strengthen information systems, evidence and research for mental health’ [2] (p. 34).

The participant data indicate that MHSW offers ‘leadership and governance for mental health’ (WHO 2013, p. 34) [2] in a variety of ways through upholding human rights and challenging others, providing leadership in relation to giving due consideration to the social determinants of health, advocacy and championing social justice.

One example is the upholding of human rights alongside bringing a social perspective to the business of involuntary treatment/hospitalisation, but conversely, this may have diluted the non-statutory role of MHSW. This was evident through the data relating to the AMHP and equivalent roles. This is also reflected in the language used, which veered from the very procedural (Scotland, England) to the more therapeutic (Ireland). It also mirrors research into statutory MHSW in England, which found that its social work participants ‘rolled over’ when working as AMHPs, meaning that they tended to follow procedural language (Vicary et al. 2019) [13].

As might be anticipated, the legal and policy frameworks differed across jurisdictions, meaning that the role of MHSW in governance was not universal. In fact, there is little that unifies mental social work across jurisdictions from a law and policy perspective. The exception, of course, is the provisions of the Council of Europe’s European Convention of Human Rights and the United Nations (UN) Convention on the Rights of Persons with Disabilities (CRPD) [19]. These are two areas where, if the jurisdiction is a member of each institution, there is a legally binding relationship. Both institutions’ provisions are natural bedfellows for social work more broadly, and MHSW more precisely, given that the focus relates to supported decision-making (Article 12 CRPD) and involuntary admission for those identified as being of ‘unsound mind’ (Art. 5 ECHR). However, the recognition of CRPD was surprisingly absent from respondents’ answers compared with the recognition of the impact of human rights law, highlighting a gap in high-level legal/policy developments and knowledge on the ground. This is interesting and perhaps surprising given the widespread and highly influential debate the UNCRPD has sparked about the ethics and legality of involuntary treatment and admission, and the significant impact it has had in requiring signatory countries to review and—in many cases—revise their mental health and capacity laws [20].

There is an argument that for MHSW to be an effective part of a ‘comprehensive, integrated and responsive mental health and social care services in community-based settings’ it needs to be better defined within a European context and therefore needs a broader definition than the one derived from the authors’ UK context [2] (p. 34). However, based on our data, the role of MHSW appears to be relatively poorly defined in any event, apart from the legally mandated AMHP of England and Wales, and the MHO of Scotland. There is no consensus on progress towards improving this, as indicated by the nuanced response from one English respondent, citing a lack of progress and commitment, in contrast to an Irish SW manager’s comments about a developing network of MHSW practitioners with associated guidance. Furthermore, in some jursidisdictions, the more defined roles appear to rise out of legally mandated functions relating to involuntary admission to hospital, rather than care, support and intervention in the community. In addition, there was a strong narrative that despite the distinctive contribution that is made by mental health social workers, there was a feeling of powerlessness compared with other professionals in a medically-dominant setting and working in an environment of cutbacks and insufficient resources. For there to be good integration, each part of the service needs to understand the other and participants felt that they experienced resource challenges and professional/status barriers that hindered their work. Therefore, greater recognition of the function of mental health social workers may be gained if the role of MHSW can be better defined. While the data suggest that some participants felt their strength was in their adaptability, phrases such as ‘jack of all trades’ may also reflect a lack of a defined role and a requirement to adapt.

There was a strong indication in the data that in addressing social determinants, MHSW does seek to ‘implement strategies for promotion and prevention in mental health’ [2] (p. 34), albeit at a micro level, and often within poorly resourced mental health systems and with inadequate legal, policy and educational frameworks. The work that MHSWs were engaged in was varied but it did appear unified in what they were seeking to achieve. Therefore, perhaps MHSW is better defined by what it aims to achieve rather than the individual tasks it performs. Some jurisdictions have legally mandated roles relating to compulsory care, whereas others have more generic welfare functions. In developing this debate, an important step will be to find out what citizens in differing jurisdictions want from social workers in mental health settings, including the actions and targets for change that promote recovery and social justice.

In terms of the final objective of the WHO 2013–20 Vision, ‘to strengthen information systems, evidence and research for mental health’ this study offers some valuable insights, particularly in relation to researching and articulating the contribution made by mental health social work and the significant gaps that remain in qualifying this [2] (p. 34). For example, such information would be important for developing a shared sense of the value of bringing more socially orientated perspectives to addressing the micro and systemic challenges people with mental health issues face. These insights should, however, be read within the study’s limitations noted below, including that some jurisdictions are represented by single respondents. However, we still chose to include their responses in the results because, as far as we are aware, it is the first explorative study to consider the role and aims of MHSW across jurisdictions; other studies have looked at the delivery of mental health services more broadly and inter-disciplinary contributions [20]. Our recommendations for future research begin with a more comprehensive mapping of the contributions of MHSW to mental health services across Europe, involving a greater number of jurisdictions and providing more detailed evidence and examples of its roles and impact. In tandem, it is important to strengthen current networks and build others to provide meaningful opportunities for knowledge exchange between practitioners and researchers. This would include a focus on the provision of education for MHSW and how initiatives might be replicated in other jurisdictions. As noted, there is also a significant gap in relation to the views of people who use mental health services and what they value in the MHSW role.

### Limitations of the Study

The survey was distributed in the English language only. This may have resulted in lower response rates from countries where English is not the first language. Similarly, some words and phrases may not directly translate and may have been misinterpreted and consequently attention was paid to any ambiguities in responses during data analysis. The authors acknowledge that the design of the survey was based upon a UK perspective. However, the survey questions were sufficiently open for participants to elaborate freely, drawing on their specific jurisdictional context. Some of the questions included prompts which thereafter were used in some of the responses to the questions by participants. The study was unfunded and therefore lacked resources that may have facilitated participants from a greater number of jurisdictions taking part; as indicated, ten European jurisdictions were represented and for six out of these there two or fewer respondents.

## 6. Conclusions

Although it is difficult to directly compare MHSW within and between jurisdictions, this study has nonetheless started to identify and explore similarities and differences in a bid to stimulate and inform the consideration of potential developments. It confirms that MHSW performs important roles but that this is set against a background of problems with resourcing, professional status, identity and role definition, and in many jurisdictions, inadequacies regarding educational provisions and legal and policy frameworks. The ideas and concepts emerging from this study are aimed at building connections and cooperation across the field of MHSW in Europe. Specifically, it outlined four main themes, relating to role; law, policy and education; distinctive contributions made by MHSW; and key challenges facing MHSW. The next steps include undertaking more comprehensive and representative investigation relating to the role of MHSW role, building research and practitioner knowledge-exchange networks and exploring what people who use mental health services believe to be valuable about MHSW and how it might be developed.

## Data Availability

Data maintained securely via authorship team.
